# ScreenSifter: analysis and visualization of RNAi screening data

**DOI:** 10.1186/1471-2105-14-290

**Published:** 2013-10-03

**Authors:** Pankaj Kumar, Germaine Goh, Sarawut Wongphayak, Dimitri Moreau, Frédéric Bard

**Affiliations:** 1 Institute of Molecular and Cell Biology, 61 Biopolis Drive, Proteos 138673, Singapore; 2 Department of Biochemistry, National University of Singapore, 8 Medical Drive, Singapore 117597, Singapore

**Keywords:** RNAi screening, Data visualization, Database, Data analysis, Data mining

## Abstract

**Background:**

RNAi screening is a powerful method to study the genetics of intracellular processes in metazoans. Technically, the approach has been largely inspired by techniques and tools developed for compound screening, including those for data analysis. However, by contrast with compounds, RNAi inducing agents can be linked to a large body of gene-centric, publically available data. However, the currently available software applications to analyze RNAi screen data usually lack the ability to visualize associated gene information in an interactive fashion.

**Results:**

Here, we present ScreenSifter, an open-source desktop application developed to facilitate storing, statistical analysis and rapid and intuitive biological data mining of RNAi screening datasets. The interface facilitates meta-data acquisition and long-term safe-storage, while the graphical user interface helps the definition of a hit list and the visualization of biological modules among the hits, through Gene Ontology and protein-protein interaction analyses. The application also allows the visualization of screen-to-screen comparisons.

**Conclusions:**

Our software package, ScreenSifter, can accelerate and facilitate screen data analysis and enable discovery by providing unique biological data visualization capabilities.

## Background

RNA interference (RNAi)-based high-throughput screening has become an increasingly important and popular approach to dissect biological pathways through reverse genetics [1,2]. RNAi is a conserved biological phenomenon through which gene expression can be silenced by the endogenous cellular machinery at the level of individual transcripts, with specificity conferred by the sequence of double-stranded RNA (dsRNA) or small-interfering RNA (siRNA) [3].

Following completion of genome sequencing of the human and model organism, it became possible to systematically screen at the genome level, and this has indeed been applied to investigate numerous biological questions and cell-based processes, with novel insights revealed for apoptosis, virus infection, membrane trafficking, and the cell [4]. To date, two main screening modalities have been developed: pooled and arrayed screens. In arrayed screens, each gene is targeted individually by at least one reagent. Given that the human genome contains approximately 22,000 protein-coding genes, a genome-wide screen generates a relatively large dataset. The analysis of these datasets has drawn inspiration from small molecule screening in terms of data quality control, normalization approaches and the definition of significance threshold; i.e. hit identification. RNAi screening results are also gene-centric; therefore, hits can be linked to prior biological pathway or protein network information.

The currently available software applications to analyze RNAi screen data, however, usually lack the ability to visualize associated gene information in a dynamic fashion (Table 1). Here, we introduce ScreenSifter, an open source desktop application for the convenient implementation of sequential, user-friendly and exhaustive analyses of RNAi screening results. Biologists with no extensive bioinformatics knowledge can upload their screen data in a simple .csv format, and have access to multiple screen analysis tools, including quality control, normalization and hit selection, as well as the ability to visualize the distribution of hit genes and graphically compare replicates. This application also facilitates comparisons between different screens. ScreenSifter has visualization tools to plot subsets of screen data (specific genes or gene groups) and provides Gene Set Enrichment Analysis (GSEA) and protein-protein interaction (PPI) information directly from and/or on hyperlinked plots.

**Table 1:** ScreenSifter in comparison to related tools

	**Input file**	**Metadata**	**Data**-**base**	**QC**/ **Normalization**	**Hits Identification**	**On target analysis**	**Gene Ontology**	**Protein**-**protein interaction**	**Programming language**	**Required programming knowledge**	**GUI**	**Operating system**	**Output results**
cellHTS [5]	Text file	Description files need to be loaded along with data file	No	Yes	Yes	No	Yes	No	R package	Yes	No	Windows, Linux, Mac (R command)	HTML output,
RNAither [6]	Text file		No	Yes	Yes	No	Yes	No	R package	Yes	No	Windows, Linux, Mac (R command)	HTML output
Screensaver [7]	Excel workbooks	Yes	Yes	No	No	No	No	No	Java	No	Yes	Cross-platform	Excel workbooks, SD files
HTSanalyzeR [8]	Pre-processed data from cellHTS2	No	No	No	No	No	Yes	Yes	R package		No	Windows, Linux, Mac (R command)	Figures,
													HTML tables
ScreenSifter	CSV file	Customized Project, Screen and Table data description through GUI	Yes	Yes	Yes	Yes	Yes	Yes	Python, wxPython	No	Yes	GUI: Windows, Mac	Tab-delimited text file, visualization plots
												Source code: Linux	

## Implementation

ScreenSifter has been developed using Python, wxPython and MySQL/SQLite3. The Python source code and executable for Windows are available on SourceForge. User Guide and examples are available at http://www.screensifter.com.

ScreenSifter uses SQLite3 with a custom-designed, normalized relational database modeled to store all screen data and data processing. Data for Project, Screen, and Screen Data is stored in separate tables that are connected in the relational database model; this organization can cater for future growth without breaking the database design and allows rapid retrieval of information. A Screen Data Table in ScreenSifter contains a unique record for a Screen, which stores the values of individual wells of specific plates. It also has Project ID, Screen ID, and Screen Data Table ID. When a Screen Data Table of x number of records (number of plates multiplied by numbers of well in each plate) is uploaded, deleted, or derived, the Screen Data Table grows or reduces for x number of records. Project, Screen and other tables inside the database are then updated accordingly.

ScreenSifter has several functions for normalization, visualization, filtering, hit identification, and biological data mining, many of which are relatively specific to RNAi screening. For example, four different normalization methods are provided: Log, Z score, B score and By Control normalizations [9,10]. This allows users to select the best-suited method for their own data and thus minimize the impact that systematic errors may have on hit selection.

In sample-based normalization, the Z score is calculated for each record using the following formula:

$$Z=\left(x_{i}-\bar{x}\right)/\sigma_{x_{i}}$$

Where *x*_*i*_ is the value of the well *i*, $\bar{x}$ is average of the values of all the wells per plate and σ_*xi*_ is the standard deviation of values of the wells per plate.

The Control normalized score is calculated using the following formula:

$$\mathrm{Control}\;\mathrm{Normalized}\;\mathrm{Score}=\frac{x_{i}-{\bar{x}}_{p}}{{\bar{x}}_{p}-{\bar{x}}_{n}}$$

Where *x*_*i*_ is the value of well *i*, ${\bar{x}}_{p}$ is the average of the values of wells of the user-specified positive controls per plate and ${\bar{x}}_{n}$ is the average of the values of wells of user-specified negative controls per plate. If the user selects only the positive or only the negative control, the formula reduces to fold change e.g.

$$\mathrm{Fold}\;\mathrm{Change}=\frac{x_{i}-{\bar{x}}_{p}}{{\bar{x}}_{p}}\;\mathrm{or}\;\mathrm{Fold}\;\mathrm{Change}=\frac{x_{i}-{\bar{x}}_{n}}{{\bar{x}}_{n}}$$

B Score is calculated for each record using the following formula:

$$B\;\mathrm{Score}=\frac{R_{\mathrm{ijp}}}{\mathrm{MA}D_{p}}$$

Where

$$R_{\mathrm{ijp}}=X_{\mathrm{ijp}}-F\left(X_{\mathrm{ijp}}\right)=X_{\mathrm{ijp}}-\left(\bar{X_{p}}-\mathrm{Median}\left(R_{i}\right)-\mathrm{Median}\left(C_{j}\right)\right)$$

X_ijp_ is the measured value in the well in row i, column j and plate p, and F(X_ijp_) is the value fitted by two-way median polish that estimates systematic measurement offsets for each row i. Median (R_i_) is the median of row i and Median(C_j_ ) is the median of the column j.

There are multiple functions for interactive visualization of biological data; i.e., Gene Set Enrichment Analysis using the GO database and protein-protein interaction network.

GSEA is calculated using Fisher’s Exact test on a 2×2 contingency table for each GO category:

It returns the odds ratio and p-value. Based on the user-selected p-value threshold, GO categories are plotted as Bar Charts upon Rectangular Selection of genes on the plot. The genes selected form the basis of this contingency table (Table 2). These genes are searched in the GO database and for each GO category, and the p-value is calculated using Fisher’s Exact test. In Table 2, x is the number of genes selected in the rectangular area on the plot; g_c_ is the total number of user-selected genes in a particular GO category; G_c_ is the total number of genes in a particular GO category; and 22,000 is the total number of genes in the genome.

**Table 2:** GSEA calculation contingency table

	**User selected genes from screen**	**In genome**
In GO category	g_c_	G_c_
Not in GO category	x - g_c_	22000 - G_c_

**Figure 1:**
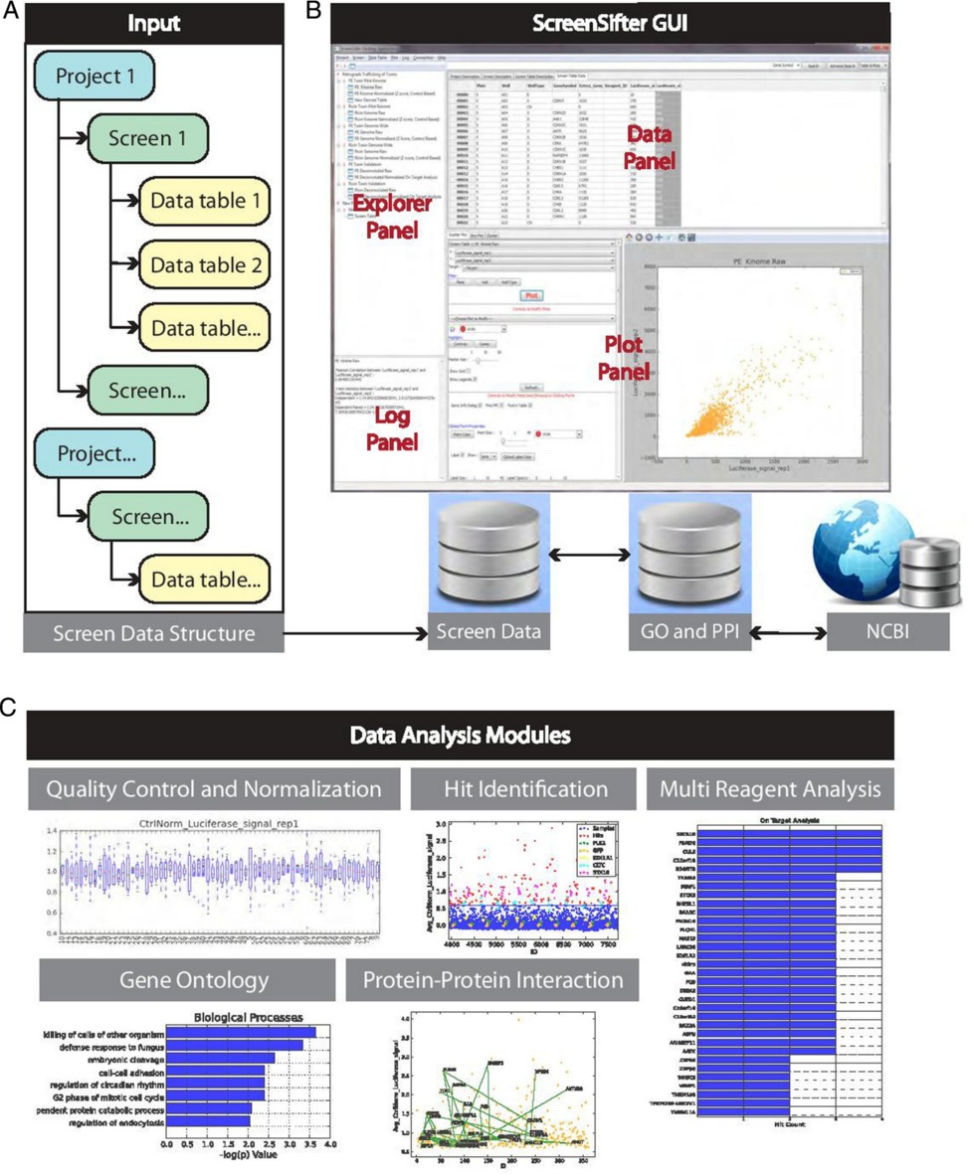
**ScreenSifter overview. (A)** Data structure in Explorer Panel, **(B)** Graphical User Interface (GUI) and database connection, and **(C)** Main data analysis modules.

The PPI function is available for a single-clicked gene or for a list of genes chosen by Rectangular Selection on the plot. If a point is clicked on the dense cloud of points, ScreenSifter first captures the nearest point based on the xy coordinates of the points. It then makes a pair of points with each other point on the plot, and each pair of points (genes) is searched in the PPI database. If a pair of genes is found in the database, then a line is drawn to connect them, indicating a PPI (Figure 1C). Similarly, for Rectangular Selection of genes, all possible combinations of two genes in the list are searched in the database and the PPIs found are highlighted by connecting lines on the plot. Additionally, if Cytoscape is open and a connection to it is enabled within ScreenSifter through the Cytoscape-RPC plugin, while creating interactions on the plot, ScreenSifter also creates a network of the same interacting points in Cytoscape.

The function called Multi Reagent Analysis lets the user analyze genes targeted by multiple RNAi reagents in different wells. When a user applies this function on any Data Column, ScreenSifter makes a sorted plot of this Data Column. When the user then sets a threshold on this plot interactively, all the RNAi reagents (Reagent ID Column in ScreenSifter Data Panel interface) that pass the threshold are mapped to their target genes. For each of these mapped genes, the number of Reagents passing the threshold is counted, and the genes are plotted in descending order. This method provides a quick, objective way of validating potential hit genes, and is a common practice in RNAi screening to validate genes of interest [11,12].

ScreenSifter also provides Z^’^ factor calculation through the Quick Analysis function; this provides a good indication of the separation of positive and negative controls in the screen. It is calculated using the following formula:

$$Z’\;\mathrm{factor}=1-\frac{3\left(\sigma_{p}-\sigma_{n}\right)}{\left|\mu_{p}-\mu_{n}\right|}$$

Where *σ*_*p*_ is the standard deviation of the positive controls, *σ*_*n*_ is the standard deviation of the negative controls, *μ*_*p*_ is the mean of the positive controls and *μ*_*n*_ is the mean of the negative controls.

ScreenSifter uses a local database for GO and PPI analysis. The database can be updated automatically by connecting to NCBI from ScreenSifter through a single click. During updating, important fields are indexed for faster retrieval.

## Results and discussion

In ScreenSifter, data is organized first by Project, which can house multiple screens and is usually defined by a specific biological question. Each Screen is defined as a specific screening experiment, corresponding to specific physical plates and including replicates. A primary data file is uploaded for each Screen and forms a primary Data Table; subsequently derived Data Tables can be saved under the same Screen. Projects, Screens, and Screen Tables are presented hierarchically in ScreenSifter (Figure 1A).

In addition to Data Tables, each Project, Screen, and Screen Table can be linked to descriptive metadata. The Project Description would specify project name, the biological question being addressed and the general experimental strategy used; an image file summarizing the project can also be uploaded. The Screen Description can contain specific information about the assay and reagents used, including siRNA library, species, and cell line(s). The Screen Table description specifies its name in ScreenSifter and the uploaded file name, its creation date, its nature (raw or derived) and, if derived, its parent table as well as a log of its derivation.

In addition to the data uploaded by users, ScreenSifter stores gene ontology and protein-protein interaction data retrieved from the NCBI websites on all human and mouse protein coding genes (Figure 1B).

The graphical user interface (GUI) is composed of four panels: the Explorer Panel allows navigation of the data structure; the Data Panel displays all metadata and data from Data Tables; the Plot Panel houses up to four plots simultaneously and includes a Plot Control Panel that allows customization; and the Log Panel displays the actions executed in ScreenSifter, as well as any results associated with the actions (Figure 1B).

The application contains several RNAi screen specific workflows, such as a Quality Control (QC) and Normalization module; a Threshold/Hit Definition module; a Multi Reagent Analysis module for the comparison of multiple siRNAs targeting the same gene and the elimination of off-target effects; and finally Gene Ontology and Protein-Protein Interaction modules for biological data mining (Figure 1C).

### Demonstration datasets

We highlight some analytical capabilities of ScreenSifter using datasets from genome-wide RNAi screens on the intracellular traffic of ribosomal-inactivating toxins in mammalian cells [13]. The Pseudomonas exotoxin A (PE) and Ricin proteins are unable to cross the plasma membrane. To reach their cytosolic targets, these toxins hijack the cells’ retrograde membrane traffic processes and, after endocytosis, move from endosomes to the Golgi complex and then to the endoplasmic reticulum (ER) where they can translocate to the cytosol and inhibit their ribosomal targets, causing inhibition of protein translation and eventually cell death [14].

**Figure 2:**
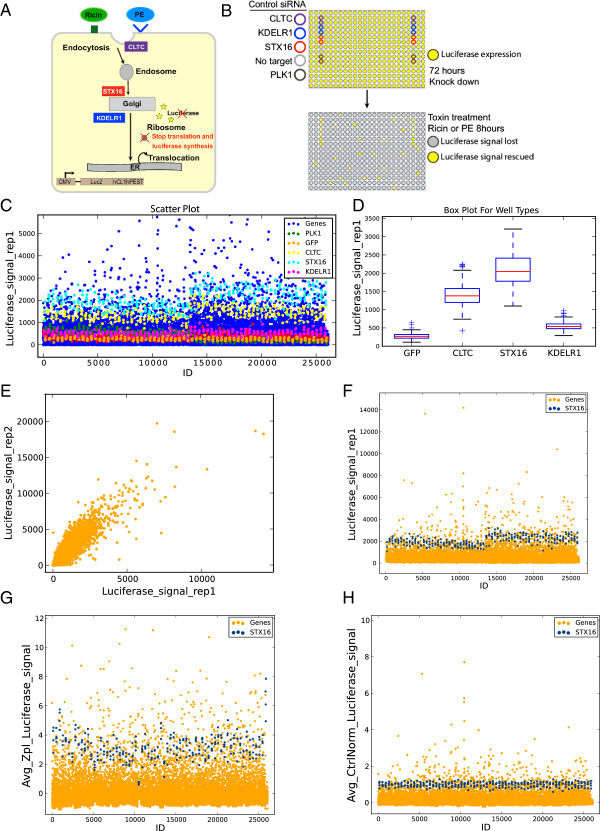
**Screen quality control and normalization. (A)** Overview of intoxication assay represented in the demonstration dataset. HeLa cells stably expressing a short half-life luciferase (Luc2CP) were treated with PE or Ricin toxins. After binding at the cell surface, both toxins are internalized into endosomes and transported to the Golgi and then to the ER, where they translocate to the cytosol. Both toxins then inhibit translation and luciferase production. The three highlighted genes, CLTC1, STX16, and KDELR1, act on endocytosis, transfer to the Golgi, and Golgi to ER traffic, respectively. **(B)** Workflow of the genome-wide screen: Cells were seeded in 384-well plates pre-printed with siRNA for reverse transfection and incubated for 72 hr, then challenged with PE or Ricin for 8 hr before luciferase signals were measured. **(C,D)** Scatter Plot, with controls highlighted **(C)**, and Box Plot of controls **(D)** of Replicate 1 of raw PE luciferase signal using the Quick Analysis module. **(E)** Plot of Replicate 1 versus 2 of raw PE luciferase signals. **(F)** Scatter Plot of Replicate 1 of raw PE luciferase signal with STX16 positive control highlighted. **(G)** Average Z-score-normalized PE luciferase signals with STX16 highlighted. **(H)** Average control (STX16)-normalized PE luciferase signals with STX16 highlighted.

The aim of these screens was to identify and compare human host genes required for PE and Ricin intoxication. To measure the capacity of either PE or Ricin toxin to reach their cytosolic target, protein synthesis was measured using a short half-life firefly luciferase (Figure 2A). HeLa cells stably expressing the luciferase were treated with siRNAs from a library consisting of 21,121 siRNAs. After 3 days, either PE or Ricin toxin were applied to the cells for 8 hours, and luciferase levels measured using luminescence. Luciferase expression thus served as a measure of the integrity of the retrograde pathway and knockdown of an important gene would result in a higher luminescence reading than that in the wild type cells (Figure 2B).

The library (siGENOME SMARTpool, Thermo Fisher) was arrayed in 384-well microplates, in which each well contained a pool of four different siRNA sequences targeting one unique gene and the two toxin screens were run in duplicate. Three positive controls consisted of siRNA targeting membrane trafficking regulators: the SNARE Syntaxin16 (STX16), the Clathrin heavy chain (CLTC) and the KDEL-Receptor 1 (KDELR1). Other controls were an siRNA targeting the Polo-Like Kinase 1 (PLK1), which induces extensive cell death and the green fluorescence protein (GFP) (Figure 2B). Further details of the screening procedure are described elsewhere [13]. The data used here is provided in the downloadable ScreenSifter package (http://www.screensifter.com).

To demonstrate the utility of ScreenSifter in handling more complicated data such as those from high-content screens, we also include in the ScreenSifter package a dataset from a high-content screen of Golgi morphology [15]. A tutorial demonstrating manipulation of this dataset is included in the software (Help > ScreenSifter Help) and on our website. It guides the user through filtering of multiple features of high-content screen data to identify hits, compiling a table of hits based on multiple features, and visualizing the hits using the hierarchical clustering plot function in ScreenSifter.

### Data upload and export capabilities

A primary .csv data file can be uploaded for each screen and will form a primary Data Table. This primary data table must contain the following fields: Plate number, Well, Well type, Gene symbol, and Entrez Gene ID. For some analyses, such as Multi Reagent Analysis, a Reagent ID entry is also required and up to 100 data columns can be included. Table Columns can be mathematically manipulated or analyzed by clicking on the respective column header. Data Tables can be exported or saved as a new Derived Table by right-clicking on the top-left corner. Undo/redo options are also available in this menu.

With the example dataset, we uploaded one .csv file for each toxin screen, each containing raw luminescence signals from the two replicates. Derived Tables were then created from these, containing normalized and averaged data. Guidance on how to upload data and save screen information is available in the help file of the software.

### Screen quality control and normalization

The raw data can first be assessed using the “Quick Analysis” module, which automatically generates scatter and box plots (Figure 2C, D), as well as a Z’ factor for all pairwise combinations of positive and negative controls. The Z’ factor is a common metric used to evaluate the quality of an assay with given positive and negative controls [16].

The Quick Analysis Scatter Plot highlights the distribution of controls among all screening wells. In our example dataset, this operation revealed a good separation between the STX16 positive control and the GFP negative control (Figure 2C). The separation is also readily observable in the box plot arranged by Well Types (Figure 2D).

To assess the reproducibility of the screens, the raw relative luminescence readouts of both replicates can be plotted, revealing a high Pearson correlation coefficient of 0.92 for both toxins (Figure 2E). This coefficient and associated t test statistics can be found in the Log Panel. In the Plot Panel, the data used for each plot is indicated on the plot by the title, which specifies the Screen Table used, and the axes labels, which correspond to the column titles.

The scatter plot also revealed significant variation among the control values across all plates of the screen (Figure 2C, F). This common phenomenon in large-scale screens requires data normalization to be applied. Visualization of whole plate-based z-scores revealed significant fluctuation in STX16 z-score values across the different plates (Figure 2G). This was found to be due to an inordinate number of outliers in some plates deriving from the non-random organization of the siRNA library. The use of a control-based normalization instead resolved this issue (Figure 2H) and from this point on, analyses were done using control (STX16)-normalized values.

### Hit identification

**Figure 3:**
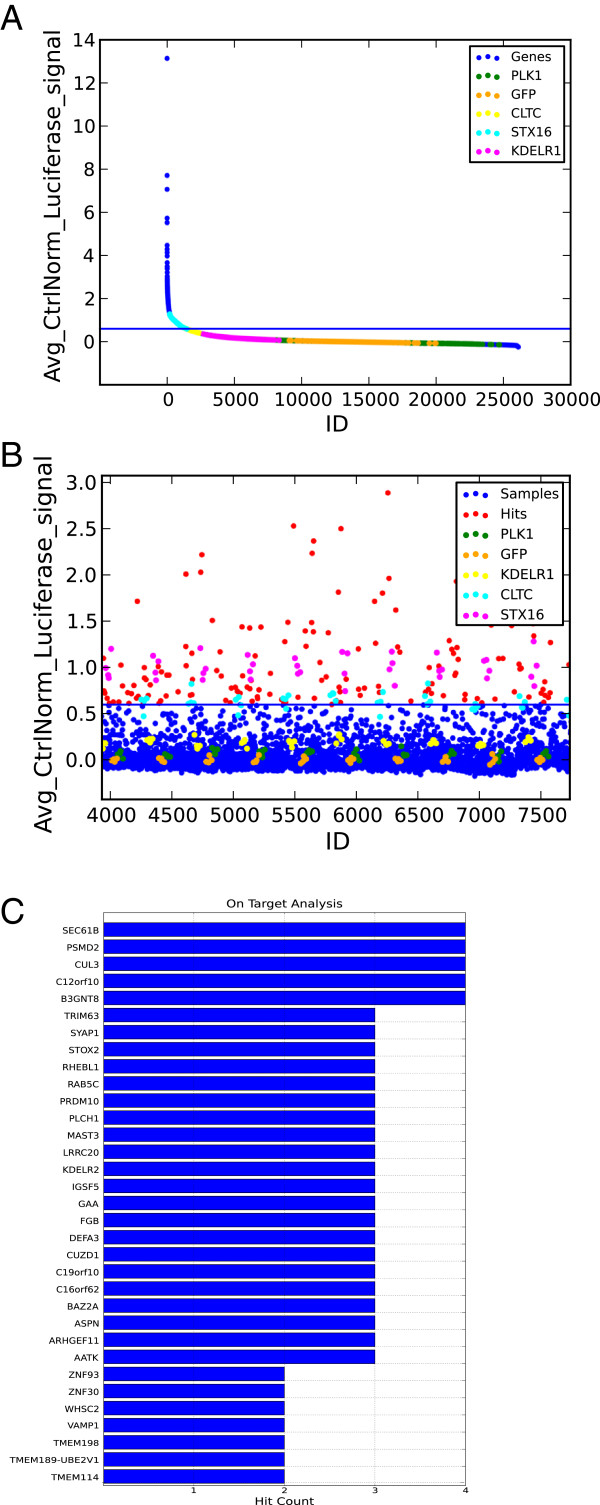
**Screen hit identification. (A)**. Threshold selection for primary hit identification on a ranked plot of the average control-normalized luciferase signal of PE. **(B)** Scatter plot of average control-normalized luciferase signal of PE, with threshold indicated and hits above threshold identified. Controls are highlighted by color codes. **(C)** Multi Reagent Analysis of PE hits: top genes ordered according to the number of validated siRNA reagents.

To determine a threshold for hit identification, ScreenSifter allows different ways to determine a cut-off using the Select Cut-off module. The module offers the possibility to use the average value and standard deviation of the samples or any controls to define a cut-off (Figure 3A). A simple cut-off can also be selected by clicking on a point in the ordered plot, or by directly entering a cut-off value. This approach is commonly used by screeners and is easy to implement. The module allows the user to test different combinations and to rapidly visualize hit lists.

The Visualize With Current Cut-off function generates a scatter plot of the data with the threshold, as well as a list of genes above the cut-off (Figure 3B). The Finalize Threshold function then generates a new column in the Screen Table, scoring each gene as a hit (1) or not (0). In both the PE and Ricin screens, the cut-offs used were values ≥60% of the average STX16 control. This also approximates the average value for the CLTC intermediate positive control (Figure 3B). After setting a threshold for each toxin and removing genes with any GO annotations containing the term “proteasome” (because these have a direct effect on the assay that does not reflect membrane trafficking events), over 2000 genes were identified to be potential significant regulators of either toxin’s trafficking.

### Hit validation by multi reagent analysis

To test a subset of genes for the possibility of off-target effects, the top 200 hits in each screen were selected and re-tested using four individual siRNAs instead of a pool. Because individual siRNAs tend to be less potent than pools, validated individual siRNAs were defined as those having a threshold signal of 30% of that of the STX16 pool, and validated genes as those having at least two validated individual siRNAs. The Multi Reagent Analysis module was applied on the validation datasets (PE Deconvoluted Raw and Ricin Deconvoluted Raw), with a 0.30 cut-off, to determine the number of validated genes. The module generated a plot of genes ranked according to the number of validated individual siRNAs (Figure 3C), as well as a list of the number and identity of validated siRNAs for each gene. The Finalize Threshold function created new columns in the Data Table: the first identifying each siRNA as validated (value “1”) or not (“0”), and the second specifying the number of validated siRNAs for that gene.

### Biological data mining

**Figure 4:**
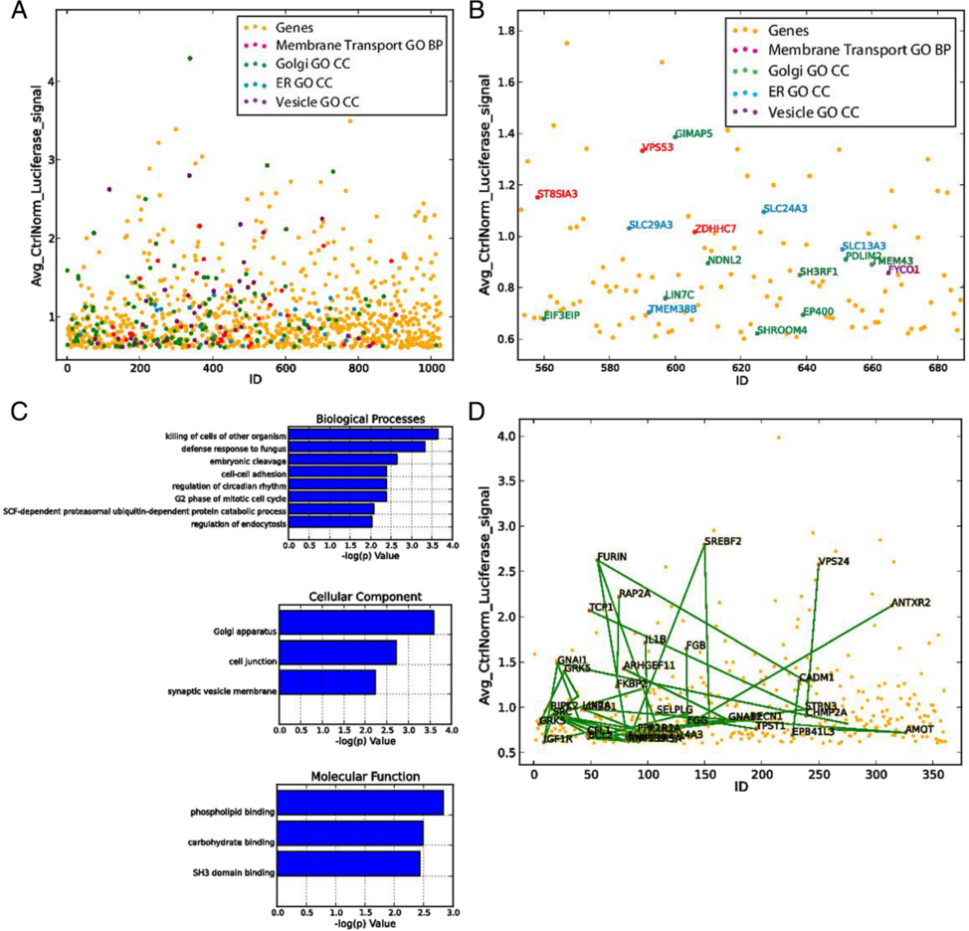
**Screen biological data mining. (A)** A search in the Plot for various GO terms highlights the relevant genes in the plot. **(B)** Zoom of the same Plot in **(A)**, with the gene labeling option enabled. **(C)** GO enrichment analysis of validated hits showing significant enrichment (p < 0.01, Fishert. exact test) of the hits in membrane trafficking-related categories. **(D)** Highlighting of genes (black labels) having protein-protein interactions (PPIs; green lines) among genes with genes with GO annotation.

The toxins we tested must undergo membrane traffic before they can intoxicate cells. Thus, to test if this process is relevant in our hit list, we used the search function in the Data Table by searching the term “membrane transport” in “GO BP (Biological Process)”, which highlighted the results in the Data Table and the current Plot (Figure 4A). We also mined the subcellular localizations of genes using more specific search terms such as “Golgi”, “ER” or “vesicle” in “GO CC (Cellular Compartment)” (Figure 4A). By checking the Label option in the Plot Control Panel, genes could be identified directly on the scatter plot (Figure 4B).

We also performed a Gene set enrichment analysis (GSEA) of the validated hit genes using a threshold p value of 0.01. This showed, among other results, a statistically significant enrichment of genes with an association with the Golgi apparatus (Cellular Compartment Gene Ontology) (Figure 4C), consistent with the requirement of the Golgi apparatus for PE intracellular trafficking.

To assess known relationships between genes of interest, PPIs between genes can be identified by checking the “Find PPI” option for Clicking Points, or checking the “Find PPI in Selected Area” option for Rectangular Selections in the Plot Control Panel. Each PPI and its associated information are listed in the Log Panel. We performed a PPI search using Rectangular Selection of all genes with “membrane” GO annotation to reveal the interconnectedness of these genes, with 46 unique PPIs identified among 360 unique hit genes (Figure 4D).

### Screen comparisons

**Figure 5:**
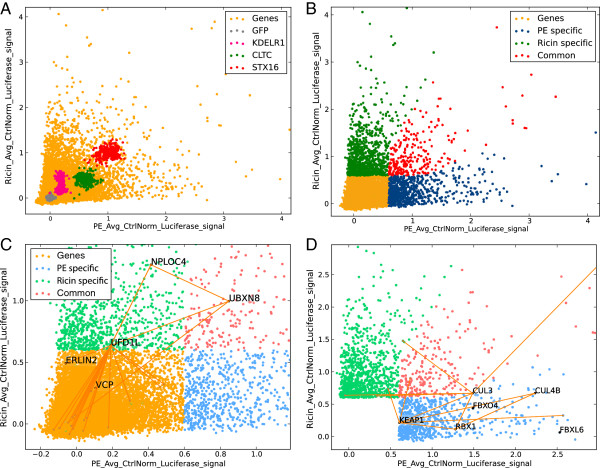
**Screen comparison. (A)** Scatter plot of average control-normalized luciferase signals of PE vs Ricin, with controls highlighted. **(B)** Scatter plot of average control-normalized luciferase signals of PE vs Ricin, color-coded according to toxin-specificity. **(C)** ERAD protein-protein interaction network displaying Ricin specificity, demonstrating the identification of a gene below but close to the chosen threshold (ERLIN2), and a potential false negative gene (VCP). **(D)** Cullin-Ring Ligase complex protein-protein interaction network displaying PE specificity.

To compare the results for the two toxins, the average control-normalized Ricin luciferase signal was plotted against that of PE (Figure 5A). The controls were highlighted on the plot using the “Highlight Controls” function in the Plot Control Panel. The plot revealed a significant divergence between the two toxins, while highlighting the remarkably consistent similar requirement for STX16 (red dots in the plot).

The toxin specificity of a gene was thereafter defined as a 2-fold difference between the two toxins. Toxin-specific gene lists were generated by using the Filter and Add/Subtract/Multiply/Divide column functions and saving the results in separate Data Tables corresponding to toxin specificities. This demonstrated that there were more toxin-specific hit genes (757 and 1058 for PE and Ricin, respectively) than common ones (262). The gene lists in these tables were then used to highlight toxin specificities in the plot by color codes, using the Highlight Genes function (Figure 5B).

Because hit threshold selection ultimately has an element of arbitrariness, and because of various experimental factors resulting in assay noise and variation, genes of significant relevance to a biological process may sometimes be narrowly missed by falling under the Hit Selection cut-off. In these situations, ScreenSifter is useful for identifying potentially important genes that may be near-threshold. This can be illustrated by Ricin-specific hits that have been linked to ER translocation (Figure 5C). ER translocation is an essential step in the traffic of PE and Ricin toxins, which have long been suspected to use the machinery of ER Associated Degradation (ERAD) to translocate. Surprisingly, some ERAD-related genes, such as NPLOC4 and UFD1L, appeared to be toxin specific. Searching for additional likely players in this process, we used PPI plotting again to identify Valosin containing Protein (VCP) and a cofactor of VCP, ERLIN2, which were below our significance threshold values. This type of finding could, for example, warrant re-testing these genes with different, perhaps more potent, knockdown reagents.

Among the genes that appeared strongly positive and toxin specific, we noticed the cullin 4B gene (CUL4B). To quickly identify partners of this protein, we employed the “PPI Display” option in the Plot Control Panel in conjunction with the Plot Search function, which revealed that ring-box 1, E3 ubiquitin protein ligase (RBX1), F-box protein 4 (FBXO4), F-box and leucine-rich repeat protein 6 (FBXL6), kelch-like ECH-associated protein 1 (KEAP1), Cullin 3 (CUL3) and F-box protein 31 (FBXO31) were present in the dataset of PE-specific hits, and are well-connected by PPIs (Figure 5D). All these proteins have been shown to interact in Cullin-Ring Ligase (CRL) complexes. These multi-subunit ubiquitin ligases are known to regulate various aspects of cell physiology but had not been previously implicated in retrograde traffic. The identification of several PE-specific subunits reinforced and highlighted their functional importance for PE trafficking.

## Conclusions

Through these examples, we have illustrated the main features of the ScreenSifter application. As a dedicated application to RNAi screening, ScreenSifter facilitates rapid and intuitive quality control for the analysis of screen data. As a desktop application working with downloaded biological databases, ScreenSifter allows a very interactive interplay between the screener, their screening dataset and publicly available gene-centric data. This flexibility and a user-friendly visual interface will favor a quick and iterative process of data exploration, with the ability to rapidly generate customized tables and graphs for reports and publications.

## Availability and requirements

**Project name:** ScreenSifter

**Project home page:**http://www.screensifter.com

**Operating system(s):** Win 7, Win XP, Mac, Linux

**Programming language:** Python, wxPython, MySQL/SQLite3

**Other requirements:** Please cite this publication if used for data analysis and figure generation

**License:** GNU GPL

**Any restrictions to use by non-academics:** None Files (installers and source code) are available for download at: http://sourceforge.net/projects/screensifter/. For each version the executable file is provided with or without database. The executable with database has preloaded databases of Gene ontology, protein-protein interaction from NCBI. (ftp://ftp.ncbi.nlm.nih.gov/gene/GeneRIF, file name interactions.gz). ScreenSifter software will work without the biological database as well. If you download ScreenSifter without database, you can add the database anytime directly from ScreenSifter under Connection -> Update ontology and Interaction database. Downloading and indexing the database may take a few hours. The datasets included in the software are from genome-wide RNAi screens on the intracellular traffic of ribosomal-inactivating toxins in mammalian cells [13] and a high-content kinome-wide RNAi screen of Golgi morphology [15].

## Abbreviations

csv: Comma separated values; dsRNA: double-stranded RNA; ER: Endoplasmic reticulum; ERAD: ER-associated degradation; GFP: Green fluorescence protein; GO (BPCC): Gene ontology (biological process, cellular component); GUI: Graphical user interface; PE: Pseudomonas exotoxin A; PPI: Protein-protein interaction; QC: Quality control; RNAi: RNA interference; siRNA: small interfering RNA.

## Competing interests

The authors declare that they no competing interest.

## Authors’ contributions

PK and FB designed the software organization. PK wrote the code. DM, GG and SW provided feedback on the software. GG, SW and FB wrote the manuscript. All authors read and approved the final manuscript.

## Authors’ information

The IMCB RNAi screening facility is run in close collaboration with the FB lab. The FB lab has run several screens and is collaborating with different groups in Singapore to perform other RNAi screens.
